# Cause-specific mortality time series analysis: a general method to detect and correct for abrupt data production changes

**DOI:** 10.1186/1478-7954-9-52

**Published:** 2011-09-19

**Authors:** Grégoire Rey, Albertine Aouba, Gérard Pavillon, Rasmus Hoffmann, Iris Plug, Ragnar Westerling, Eric Jougla, Johan Mackenbach

**Affiliations:** 1 INSERM, CépiDc, Le Kremlin-Bicêtre, France; 2 Erasmus MC, Department of Public Health, Rotterdam, The Netherlands; 3 Department of Public Health and Caring Sciences, Social Medicine, Uppsala University, Uppsala, Sweden

## Abstract

**Background:**

Monitoring the time course of mortality by cause is a key public health issue. However, several mortality data production changes may affect cause-specific time trends, thus altering the interpretation. This paper proposes a statistical method that detects abrupt changes ("jumps") and estimates correction factors that may be used for further analysis.

**Methods:**

The method was applied to a subset of the AMIEHS (Avoidable Mortality in the European Union, toward better Indicators for the Effectiveness of Health Systems) project mortality database and considered for six European countries and 13 selected causes of deaths. For each country and cause of death, an automated jump detection method called Polydect was applied to the log mortality rate time series. The plausibility of a data production change associated with each detected jump was evaluated through literature search or feedback obtained from the national data producers.

For each plausible jump position, the statistical significance of the between-age and between-gender jump amplitude heterogeneity was evaluated by means of a generalized additive regression model, and correction factors were deduced from the results.

**Results:**

Forty-nine jumps were detected by the Polydect method from 1970 to 2005. Most of the detected jumps were found to be plausible. The age- and gender-specific amplitudes of the jumps were estimated when they were statistically heterogeneous, and they showed greater by-age heterogeneity than by-gender heterogeneity.

**Conclusion:**

The method presented in this paper was successfully applied to a large set of causes of death and countries. The method appears to be an alternative to bridge coding methods when the latter are not systematically implemented because they are time- and resource-consuming.

## Background

The study of cause-specific mortality time series is one of the main sources of information for public health monitoring [1-3]. However, while demonstrative and striking use can be made of such trends when communicating with the general public, many concerns relating to the data production process have to be addressed. More specifically, it is necessary to evaluate, and, if necessary, correct artifacts due to data production changes that may bias the interpretation of time trends over a study period.

The production processes for mortality databases have been similar in many industrialized countries (particularly in Western Europe) since the end of World War II. When a death occurs, a medical certificate based on the international form recommended by the World Health Organization (WHO) [4] is filled in by a physician. The physician reports the causes of death that directly led or contributed to the death on the death certificate. The death certificate is then forwarded to a national (e.g., France) or regional (e.g., Germany) coding office, where it is coded using the International Classification of Diseases (ICD). The ICD has been regularly reviewed and improved since the end of the 19^th ^century [5]. For each death, an underlying cause of death is selected in compliance with the ICD rules. The underlying cause is the most commonly used in statistical analyses.

Underlying cause coding is a complex process and thus implies potential between-coder coding differences. These differences may produce coding discrepancies over time and space. This is why, in addition to ICD revisions, coding may induce variations in the causes of death by period, region, or country. This situation has resulted in countries using increasingly automated coding systems (ACS).

Variations in mortality trends may also be related to changes in death certification (death certificates, certification habits, diagnoses, etc.). However, the changes are often diffuse and take place over long periods of time, making them harder to take into account. Moreover, the methods of analyzing actual historical variations in mortality by cause of death and taking into account data production process changes are essentially related to changes in the coding process. Of these methods, three main kinds can be distinguished: bridge coding, concordance table and cause recombination, and time series analysis-based methods.

### Bridge coding

The bridge coding method is used when there is a major change in the coding process (ICD version change or switch from a manual to an automatic coding system). The method consists of coding a large set of death certificates twice, applying the rules prevailing before and after the change. The ratios of numbers of death calculated by cause category before and after the change, called "comparability ratios," generate information for trend analyses and characterization of "jumps" in mortality time series. However, analyses of long-period time series do not necessarily use comparability ratios [1,6].

Bridge coding analyses have been carried out in the United States and England and Wales for each ICD change since the eighth version of the ICD (ICD-8) [7-12]. Bridge coding analysis was also used and generated detailed results for the change from ICD-9 to ICD-10 in some countries (Scotland, Sweden, Italy, Spain, France, and Canada) [13-18]. However, to the authors' knowledge, most European countries have not implemented bridge coding to assess ICD changes. Comparability ratios are heterogeneous between country, most likely because of variations in intra-group composition of causes of death, reporting practices, and ICD coding interpretation. Therefore, it is unlikely that a comparability ratio for one country can be inferred from the results of other countries.

### Concordance table and cause recombination

The concordance table and cause recombination approach consists of determining the most consistent cause categories, under medical consideration, for two successive ICD revisions. Analysis of mortality using the resulting categories is then theoretically influenced little by coding changes. This approach typically only works well when considering the coding of any particular cause reported on the death certificate. It is often not effective when considering changes in the rules for selecting the underlying cause of death, especially when such rule changes favor the selection of one cause over another. It is often impossible to recombine codes to fully account for these changes.

The method was used on French, Dutch, and Swedish data [19-21]. This approach, complex and time-consuming when it is applied to a single country, is even more difficult to use in the context of an international study [22].

### Time series analysis

The time series analysis method consists of looking for sustainable jumps, evaluating their statistical significance and amplitude, and possibly smoothing the time series by adjusting the data with correction factors. The method is easy to document, even when the volume of data considered is large (many countries, many causes of death, etc.). Furthermore, the method is necessary when the time of the change in the data production process is unknown [23]. To the authors' knowledge, the detection of jumps in mortality data has rarely been undertaken [22,24], but, in particular for Janssen et al's work [22], has given rise to fruitful international public health studies [25-28]. However, the methods used in these studies did not take advantage of the recent development of automated jump detection methods in indexed data analysis (by time or other variables) [29-31]. Interest in the automatic jump detection method resides in its ability to avoid the subjectivity of visual detection or a priori selection of jump positions.

The aim of this paper is to propose a complement to a time series analysis method that was previously developed by Janssen et al. [22], allowing detection of sustainable jumps attributable to changes in data production and development of correction factors by age and gender in order to enable subsequent epidemiological analyses. The method is then applied to a wide range of different mortality time series: 13 causes of death for each of six European countries participating in the AMIEHS (Avoidable Mortality in the European Union, toward better Indicators for the Effectiveness of Health Systems) project http://amiehs.lshtm.ac.uk/.

## Methods

### General approach

The following step-by-step approach was adopted:

1. Given a list of selected causes of death, the ICD codes to be considered were determined by nosologists based on the correspondence table method, while maintaining the medical consistency of the list of codes for the various ICD revisions.

2. An automated jump detection method was applied to the mortality rate time series for each of the selected causes of death.

3. For documented jumps (e.g., ICD changes), the available comparability ratios were compared to the amplitude of the estimated jumps. For nondocumented jumps, general information feedback was requested from the national data producers.

4. For documented or plausible jump positions, the statistical significance of the between-age and between-gender jump amplitude heterogeneity was evaluated by means of a regression model, and correction factors were deduced from the results.

### Mortality data

The mortality data were derived from the AMIEHS project dataset. Six countries were included in the analysis: Spain, France, the Netherlands, Germany, Sweden, and England and Wales (considered together). In order to simplify presentation, Estonia, which is participating in the AMIEHS project, has not been considered in this paper because Estonia used a specific coding system until 1994. The study period is 1970 to 2005. While causes of death are usually coded with four-digit ICD codes, the AMIEHS dataset only contains three-digit codes for practical reasons. Some precision in the characterization of causes has thus been lost.

Generally, deaths are coded using the same ICD revision in each calendar year. The dates of the ICD revisions used by each European country are presented in table 1.

**Table 1:** Dates of ICD change and automatic coding system (ACS) implementation for six of the AMIEHS European countries

Country	ICD-8	ICD-9	ICD-10	ACS
England and Wales	1968	1979	2001	1993
France	1968	1979	2000	2000
Germany	1968	1979	1998	2008
Netherlands	1969	1979	1996	
Spain	1968	1980	1999	
Sweden	1969	1987	1997	1987

### Code allocation

For 13 causes of death selected in the AMIEHS project, the method of allocating the ICD-8, ICD-9, and ICD-10 codes was as follows:

• When the cause was included in the Eurostat 65 causes shortlist [32], the codes defined by Eurostat were retained.

• For other causes, two nosologists independently selected the optimal three-digit codes. Then, a final choice was made in order to minimize the coding-related jumps in cause of death-specific time series analysis. Table 2 shows the related codes.

**Table 2:** ICD-8, ICD-9 and ICD-10 codes for the 13 selected causes of death

Cause	ICD-8	ICD-9	ICD-10
Cerebrovascular disease	430-438	430-438	I60-I69
Conditions originating in the perinatal period	760-779	760-779	P00-P96
Congenital heart disease	746	745-746	Q20-Q24
Heart failure	428-429	428-429	I50-I51
Hodgkin's disease	201	201	C81
Hypertension	400-404	401-404	I10-I13
Ischemic heart disease	410-414	410-414	I20-I25
Malignant colorectal neoplasm	153-154	153-154	C18-C21
Malignant neoplasm of cervix uteri	180	180	C53
Malignant neoplasm of testes	186	186	C62
Peptic ulcer	531, 532	531, 532	K25-K26
Renal failure	593, 792	584-586	N17-N19
Rheumatic heart disease	390-398	390-398	I00-I09

### The automatic jump detection method

The Polydect method [33] was applied to yearly log mortality rate time series for each country and cause of death.

Given that mortality analyses are often based on multiplicative assumptions, log-linear generalized models were used. Thus, the time series jump detection method was applied to the log mortality rates.

Let O_t_, be the number of deaths during year t, p_t _be the number of person-years, and ${\text{L}}_{\text{t}}=\log\left(\frac{{\text{O}}_{\text{t}}}{{\text{p}}_{\text{t}}}\right)$ be the log mortality rate time series. The occurrence of jumps in the log mortality rate time series may be expressed as follows:

$$\begin{array}{c} \log\left(\text{E}\left({\text{O}}_{\text{t}}\right)\right)=\log\left({\text{p}}_{\text{t}}\right)\\ +\text{g}\left(\text{t}\right)+\underset{\text{t’}\in \text{S}}{\sum }{\text{d}}_{\text{t’}}\cdot 1_{\left(\text{t}>\text{t’}\right)}, \end{array}$$

In which g is a continuous function, S is the set of jump locations, and {d_t_,t ∈ S} are the corresponding jump magnitudes.

In this model, g, *S*, and {d_t_, t ∈ S} are all assumed unknown.

The method consists of three main steps:

1. A left and right limit of E(L_t_) were estimated for each point t using two local polynomial smoothers, denoted P_l_(t) and P_r_(t), fitted on [t - h, t) and (t, t + h], respectively, where h is the bandwidth for the estimation to be estimated in further steps. If t ∉ S, and the jumps location are distant from at least h, then, given that g is continuous, we expect E(P_1_(t)) = E(P_r_(t)) = g(t). Else, if t ∈ S, we expect E(P_1_(t)) = g(t) and E(P_r_(t)) = g(t)+d_t_.

The noise σof the L_t _process is estimated as:

$$\hat{\sigma }=\frac{\underset{\text{t}}{\sum }\min\left({\left({\text{L}}_{\text{t}}-{\text{P}}_{\text{l}}\left(\text{t}\right)\right)}^{2},{\left({\text{L}}_{\text{t}}-{\text{P}}_{\text{r}}\left(\text{t}\right)\right)}^{2}\right)}{\text{T}-1}$$

The polynomial kernel of the smoothers could, a priori, be constant, linear, or quadratic, depending on the number of observations and the curvature level of the time series. Since, in the present case, the number of observations was not greater than 40, and the time series was expected to be quite stable, a linear kernel was selected.

2. Considering M(t) = P_r_(t)-P_1_(t), jump points were defined as points where the signal-to-noise ratio $\frac{\left|\text{M}\left(\text{t}\right)\right|}{\hat{\sigma }}$ was higher than a threshold *C*_α_.

*C*_α _was chosen such that, if t is not a jump point, $\text{P}\left(\frac{\left|\text{M}\left(\text{t}\right)\right|}{\hat{\sigma }}>{\text{C}}_{\alpha }\right)\leq \alpha$. The analytic calculation of *C*_α _is given elsewhere [33]. In the following steps, α was set to 10^-5^, a low value, in order to avoid as many as possible false positive jumps.

Then, $\text{S}=\left\{\text{t}:\frac{\left|\text{M}\left(\text{t}\right)\right|}{\hat{\sigma }}>{\text{C}}_{\alpha }\right\}$ and $\left\{{\hat{\text{d}}}_{\text{t}}=\text{M}\left(\text{t}\right),\text{t}\in \hat{\text{S}}\right\}$ were directly estimated. When several jumps were detected in a time range less than the bandwidth, only the jump that maximized M(t) was retained.

3. The bandwidth h was estimated by minimizing the Hausdorff distance [29], defined as:

$$\begin{array}{c} {\text{d}}_{\text{H}}\left(\text{S},\hat{\text{S}};\text{h}\right)=\\ \max\left\{\underset{{\text{t}}_{\text{1}}\in \hat{\text{S}}}{\sup}\;\underset{{\text{t}}_{\text{2}}\in \hat{\text{S}}}{\inf}\left|{\text{t}}_{1}-{\text{t}}_{2}\right|,\underset{{\text{t}}_{\text{1}}\in \hat{\text{S}}}{\inf}\;\underset{{\text{t}}_{\text{2}}\in \hat{\text{S}}}{\sup}\left|{\text{t}}_{1}-{\text{t}}_{2}\right|\right\}, \end{array}$$

in which ${\text{d}}_{\text{H}}\left(\text{S},\hat{\text{S}};\text{h}\right)$ was calculated through a bootstrap procedure, setting B, the number of batches used, equal to 1000. A full description of this method is given elsewhere [33].

For a given jump i, the multiplicative factor MF_i _between before and after the jump period was calculated as:

$$\text{M}{\text{F}}_{\text{i}}=\exp\left({\text{d}}_{\text{i}}\right),$$

in which d_i _is the amplitude of the jump.

### Age- and gender-heterogeneity test

Age categories were defined as the tertile of the cause-specific death counts.

Generally, when considering J different population groups (age and gender), a generalized additive model (GAM) with an overdispersed Poisson distribution is used [34,35]. The model has the following form:

$$\begin{array}{c} \log\left(\text{E}\left({\text{O}}_{\text{t,j}}\right)\right)=\log\left({\text{p}}_{\text{t,j}}\right)\\ +{\text{g}}_{\text{j}}\left(\text{t}\right)+\underset{\text{t’}\in \text{S}}{\sum }{\text{d}}_{\text{t’,j}}\cdot 1_{\left(\text{t}>\text{t’}\right)}, \end{array}$$

in which j is one of the J groups, g_j _are continuous functions fitted by a thin plate penalized regression spline, S is the set of jump locations, and {d_t,j_, t ∉ S} are the corresponding jump magnitudes for group j.

S is supposed known, and the aim is to test for each t ∈ S:

$$\text{H0:}{\text{d}}_{\text{t},1}=\cdots ={\text{d}}_{\text{t,J}}$$

Backward variable selection was used to suppress, successively, age and gender from the model if their respective effects on the jump amplitude were not statistically significant at the 5% level, using Wald's test.

The MGCV (Multiple smoothing parameter estimation by Generalized Cross Validation) R package was used for this purpose [36].

### Correction factors

Correction factors were calculated for all confirmed jumps.

The correction factors were calculated for use in subsequent analyses, not discussed in this article, with a log-linear model of general form:

$$\begin{array}{c} \log\left(\text{E}\left({\text{O}}_{\text{t}}\right)\right)=\\ \log\left({\text{p}}_{\text{t}}\right)+{\text{c}}_{\text{t}}+\text{f}\left({\text{X}}_{\text{t}}\right), \end{array}$$

in which t is the year between T_1 _and T_2 _(respectively equal to 1970 and 2005 in this study); c_t _is the correction factor and f(X_t_) could be any function of independent variables to be estimated.

The correction factors were set so that the last values of the corrected mortality rates were equal to the exact mortality rates, i.e., ${\text{c}}_{{\text{T}}_{2}}=0$. This choice was based on the supposed superior quality and between-country comparability of the most recent year's data.

The foregoing results in the following definition of the correction factors c_t_:

For t ∈ [T_1_, T_2_],

$$\begin{array}{c} {\text{c}}_{\text{t}}=\underset{\text{t’}\in \text{S},\text{t’}<\text{t}}{\sum }{\text{d}}_{\text{t’}}-\underset{\text{t’}\in \text{S}}{\sum }{\text{d}}_{\text{t’}}\\ =-\underset{\text{t’}\in \text{S},\text{t’}\geq \text{t}}{\sum }{\text{d}}_{\text{t’}}. \end{array}$$

The estimate of c_t _was then directly obtained from the estimates of S and d_t _detailed earlier.

A corrected version of the log mortality rate was then obtained as:

$${\text{L}}_{\text{t}}^{\text{cor}}={\text{L}}_{\text{t}}-{\text{c}}_{\text{t}}$$

## Results

### Jump detection

By applying the jump detection method to all of the time series, a set of jumps was obtained (table 3). Most of the jumps detected were concomitant with a known coding change (ICD updates or change from a manual to an automatic coding system). Some of the jumps (e.g., for heart failure and rheumatic heart disease) were of great amplitude and almost systematically observed in each country. For the former East Germany, most of the changes were concomitant with the reunification of Germany.

**Table 3:** Jumps in the log mortality rate time series for the 13 selected causes of death from 1970 to 2006 identified by the Polydect method

Country	Underlying cause of death	Year of the jump	**Multiplicative factor**^**1**^	Data producer confirmation	**Proposed jump cause**^**2**^
England and Wales	Cerebrovascular disease	2001	1.24	Yes	ICD-10
	Conditions originating in the perinatal period	1986	0.09	No answer	No
	Conditions originating in the perinatal period	2001	2.78	Yes	ACS
	Heart failure	1979	3.59	Yes	ICD-9
	Heart failure	1984	0.74	Yes	No
	Heart failure	1993	1.61	Yes	ACS
	Renal failure	1979	2.90	Yes	ICD-9
	Renal failure	1993	2.33	Yes	ACS
	Rheumatic heart disease	1979	0.63	Yes	ICD-9
	Rheumatic heart disease	2001	0.77	Yes	ICD-10

France	Heart failure	1979	1.99	Yes	ICD-9
	Hypertension	1979	0.71	Yes	ICD-9
	Hypertension	1998	1.26	Yes	Death certificate
	Malignant colorectal neoplasm	1979	0.92	Yes	ICD-9
	Malignant colorectal neoplasm	2000	0.94	No	ICD-10
	Peptic ulcer	1979	0.74	Yes	ICD-9
	Peptic ulcer	2000	0.63	Yes	ICD-10
	Rheumatic heart disease	1979	0.36	Yes	ICD-9
	Rheumatic heart disease	2000	1.54	Yes	ICD-10

East Germany	Cerebrovascular disease	1991	1.45	Yes	Reunion
	Conditions originating in the perinatal period	1991	0.46	Yes	Reunion
	Heart failure	1990	1.85	Yes	Reunion
	Heart failure	1991	0.52	Yes	Reunion
	Hypertension	1991	0.51	Yes	Reunion
	Ischemic heart disease	1991	1.43	Yes	Reunion
	Malignant colorectal neoplasm	1991	1.21	Yes	Reunion
	Rheumatic heart disease	1998	1.14	Yes	ICD-10

West Germany	Heart failure	1979	1.65	Yes	IC-D9
	Hodgkin's disease	1994	0.59	No	No
	Ischemic heart disease	1979	0.87	Yes	ICD-9
	Rheumatic heart disease	1979	0.51	Yes	ICD-9

Netherlands	Heart failure	1979	1.54	Yes	ICD-9
	Heart failure	1996	0.76	No answer	ICD-10
	Hypertension	1980	0.65	Yes	No
	Renal failure	1979	0.75	No answer	ICD-9
	Rheumatic heart disease	1992	5.40	No answer	No
	Rheumatic heart disease	1996	0.13	No answer	ICD-10

Spain	Conditions originating in the perinatal period	1975	2.94	Yes	Definition change
	Heart failure	1980	1.41	Yes	ICD-9
	Ischemic heart disease	1980	0.93	Yes	ICD-9
	Malignant colorectal neoplasm	1980	0.78	Yes	ICD-9
	Renal failure	1980	1.73	Yes	ICD-9

Sweden	Heart failure	1987	5.55	Yes	ICD-9
	Malignant neoplasm of testes	1974	1.33	Yes	No
	Malignant neoplasm of testes	1979	0.74	Yes	No
	Renal failure	1987	0.69	Yes	ICD-9
	Renal failure	1997	1.23	Yes	ICD-10
	Rheumatic heart disease	1982	0.08	Yes	Coding change
	Rheumatic heart disease	1987	15.33	Yes	ICD-9

^1^: multiplicative factor of the mortality rate between the period before and after the detected jump

^2^: new coding, implementation of which may have caused the jump

The answers from data producers, contacted to determine whether the jump was related to a data production issue, were consistent between countries. Most (excluding the "No answer," 42 out of 44) of the detected jumps were confirmed to be related to a coding change. The three-digit coding constraint was given as an explanation for some jumps (rheumatic heart disease in France, ischemic heart disease in Spain, etc.), especially when countries chose specific codes (as in Spain for malignant colorectal neoplasm). The 1990 and 1991 jumps in East Germany were related to a complete change of coding staff. However, most of the coding changes are not documented by a literature reference.

Given the large proportion of confirmed jumps, we decided to exclude from subsequent treatment the jumps for which we received a negative answer from data producers.

It was possible to compare a few of the multiplicative factors with the comparative ratios generated by bridge coding studies corresponding to ICD-9 to ICD-10 changes (table 4). In particular, the large multiplicative factors (e.g., for rheumatic heart disease) had no related comparative ratios. Some coding changes were not detected by the jump detection method (Hodgkin's disease in England and Wales and Sweden and renal failure in England and Wales). However, none of the detected jumps were found unrelated to a coding change.

**Table 4:** Comparative ratios (CR) between bridge coding and multiplicative factors (MF) estimated by the jump detection method for ICD-9 to ICD-10 coding change

	France		England and Wales		Sweden		Spain	
**Underlying cause of death**	**CR**	**MF**	**CR**	**MF**	**CR**	**MF**	**CR**	**MF**

Cerebrovascular disease	0.97	1.00	1.13	1.13	1.03	1.00	-	1.00
Conditions originating in the perinatal period	1.03	1.00	-	2.24	1.02	1.00	-	1.00
Congenital heart disease	-	1.00	-	1.00	-	1.00	-	1.00
Heart failure	-	1.00	-	1.00	-	1.00	-	1.00
Hodgkin's disease	-	1.00	1.06	1.00	1.13	1.00	-	1.00
Hypertension	-	1.00	-	1.00	-	1.00	-	1.00
Ischemic heart disease	1.03	1.00	1.01	1.00	1.00	1.00	1.00	1.00
Malignant colorectal neoplasm	0.97	0.94	1.01	1.00	0.99	1.00	1.00	1.00
Malignant neoplasm of cervix uteri	0.92	1.00	1.00	1.00	1.00	1.00	-	1.00
Malignant neoplasm of testes	-	1.00	0.99	1.00	-	1.00	-	1.00
Peptic ulcer	-	0.63	1.00	1.00	-	1.00	-	1.00
Renal failure	-	1.00	1.08	1.00	-	1.23	-	1.00
Rheumatic heart disease	-	1.54	-	0.77	-	1.00	-	1.00

CR: Comparative ratios obtained from bridge coding, calculated as the ratio of numbers of death calculated by cause category before and after the ICD change

MF: Multiplicative factor of the mortality rate between the period before and after the ICD change, equal 1.00 when no jump was detected

-: CR not available

### Corrected mortality rate time series

**Figure 1:**
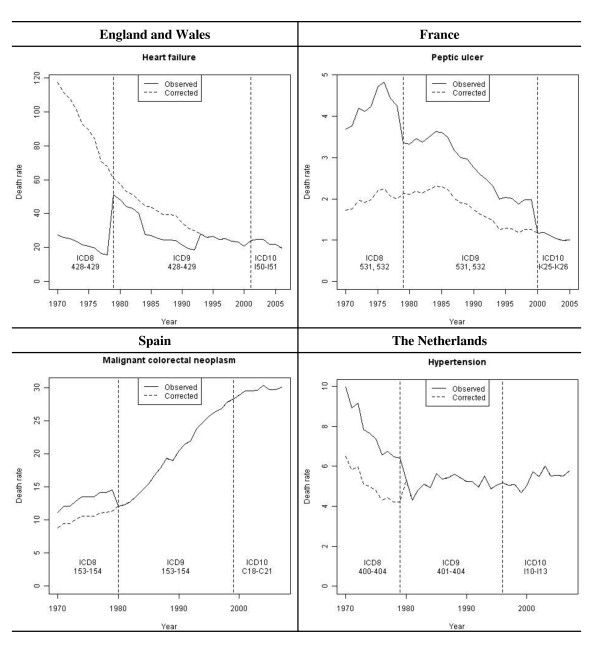
**Examples of corrected mortality rate (per 100,000 people) time series**.

Considering some of the most clear-cut time series, the profile of the corrected time series is quite different from that of the uncorrected series (Figure 1). It is noteworthy that the corrected curves do not reduce the general trends at the jump positions, which would have been the case if constant rather than linear kernel smoothing was chosen. Rather, they prolong the trends, even if the jump is in the opposite direction of the general trend.

Concerning hypertension in the Netherlands, we observe a trend shift between the periods before and after the corrected jumps. Such trend shift is not taken into account by the current method.

### Estimates of jump amplitudes by age and gender

With regard to the jump amplitude heterogeneity test by age and gender, only 19 out of 47 jumps were not statistically significantly heterogeneous (table 5). Five of the jumps were heterogeneous by gender, 15 by age, and eight by age and gender simultaneously. While the jump amplitudes are of the same order by gender, even when statistically heterogeneous, they are of different orders when considered by age group. This was particularly marked for rheumatic heart disease and heart failure.

**Table 5:** Multiplicative factor by age and gender, if statistically heterogeneous, for each detected jump

Country	Underlying cause of death	Year	**Gender**^**2**^	**Multiplicative factor**^**1**^		
				
				**Age tertile**^**3**^		
				
				1st	2nd	3rd
	Cerebrovascular disease	2001	Both	1.13		
	
	Conditions originating in the perinatal period	1986	Both	0.10		
		
		2001	Both	2.24		
	
England and Wales	Heart failure	1979	Male	4.78	3.86	3.34
			Female	4.22	3.41	2.95
		
		1984	Both	0.60	0.72	0.77
		
		1993	Both	1.83	1.58	1.37
	
	Renal failure	1979	Both	2.59		
		
		1993	Both	2.93	1.53	1.50
	
	Rheumatic heart disease	1979	Male	0.54	0.50	0.39
			Female	0.82	0.75	0.59

		2001	Both	0.87	0.94	0.75
	Heart failure	1979	Both	1.91		
	
	Hypertension	1979	Male	0.63		
			Female	0.70		
		
		1998	Both	1.14	1.10	1.25
	
France	Malignant colorectal neoplasm	1979	Both	0.91		
	
	Peptic ulcer	1979	Both	0.75		
		
		2000	Both	0.61		
	
	Rheumatic heart disease	1979	Male	0.39	0.32	0.22
			Female	0.62	0.51	0.35
		
		2000	Both	1.53		

	Cerebrovascular disease	1991	Both	1.40		
	
	Conditions originating in the perinatal period	1991	Both	0.52		
	
	Heart failure	1990	Both	1.71	1.46	1.27
		
		1991	Both	0.72		
	
East Germany	Hypertension	1991	Male	0.45	0.51	0.58
			Female	0.52	0.58	0.67
	
	Ischemic heart disease	1991	Male	1.14	1.21	1.30
			Female	1.22	1.30	1.40
	
	Malignant colorectal neoplasm	1991	Male	1.14	1.17	1.32
			Female	1.04	1.07	1.20

	Rheumatic heart disease	1998	Male	0.92		
			Female	1.25		
	Heart failure	1979	Both	1.60		
	
West Germany	Ischemic heart disease	1979	Male	0.98	0.92	0.76
			Female	0.93	0.88	0.72
	
	Rheumatic heart disease	1979	Male	0.46		
			Female	0.69		

	Heart failure	1979	Male	2.15	1.33	1.23
			Female	1.86	1.15	1.06
		1996	Both	0.64	0.77	0.82
	
Netherlands	Hypertension	1980	Both	0.95	0.81	0.70
	
	Renal failure	1979	Both	0.41	0.72	0.63
	
	Rheumatic heart disease	1992	Both	0.89	2.76	5.32
		1996	Both	0.42		

Spain	Conditions originating in the perinatal period	1975	Male	2.85		
			Female	3.08		
	
	Heart failure	1980	Both	1.46		
	
	Ischemic heart disease	1980	Both	0.94		
	
	Malignant colorectal neoplasm	1980	Male	0.84		
			Female	0.75		
	
	Renal failure	1980	Both	1.66	1.57	1.24

	Heart failure	1987	Both	3.57	7.55	7.99
	
	Malignant neoplasm of testes	1974	Both	1.40		
		1979	Both	0.41	0.72	0.88
	
Sweden	Renal failure	1987	Both	0.65		
		1997	Both	1.59	0.88	1.23
	
	Rheumatic heart disease	1982	Both	0.12		
		1987	Both	5.71	7.20	14.27

^1^: Multiplicative factor of the mortality rate between the period before and after the detected jump

^2,3^: Multiplicative factor estimated by age and gender, if statistically heterogeneous at the 5% level. Age and gender categorical variables were selected with a backward procedure.

^3^: Age categories were defined by country and cause of death in order to include a third of the number of deaths

## Discussion

The originality of the methodology reported herein mainly resides in its ability to detect jumps automatically using the Polydect method, without a priori or visual investigation for jump positions. In addition, application of the method to a large dataset is less time-consuming and less human-dependent than any other known method.

Some methodological choices were made, such as the choice of a linear kernel smoother and the choice of the probability α of detecting fake jumps. Considering a constant kernel smoother or different values of α slightly affected the final set of detected jumps and only for time series in which the jump amplitudes were of an order comparable to that of the overall noise of the time series. Choosing a low value of α insured a better accuracy in the jump's amplitude estimation, which is more statistically stable when the jump is of much larger amplitude than the overall noise of the time series. According to the visual inspection of time series graphs and comparable bridge coding results, a jump's amplitude estimates were reliable enough to be used in subsequent analyses.

The codes used in this study to characterize the conditions were not chosen to be used in all contexts. Indeed, they were allocated with the constraint of being comparable between three versions of the ICD and based on three-digit codes. Taking each ICD individually would certainly have led us to select other codes.

The method is designed to detect sustained jumps. Therefore, it is not sensitive to the occurrence of one-year outliers in time series data and it does not necessitate considering them separately, unlike other methods [22].

However, the proposed method is not able to detect and correct for nonabrupt data production changes. For example, if a new death certificate form, impacting certification practice and final coding, slowly spread through the population (as was the case in France between 1997 and 1999), the impact on yearly death counts would occur over several years. But, to the authors' knowledge, no general method is able to correct time-spread data production changes.

When comparable, the multiplicative factors obtained from bridge coding studies and time series methods were similar [11,15-17,37,38].

The purpose of this article is not to challenge bridge coding studies. However, bridge coding studies are not implemented in all countries, and it would be very difficult and costly to do so retrospectively for every data production change. The time series analysis methods proposed herein provide a reliable way of correcting data production changes affecting death count time trends.

Given the indirect manner in which data production changes are identified, the method necessitates feedback from data producers in order to confirm and explain the plausibility of the changes. Without that additional information, the automatic method would blindly correct any detected jumps, some of which may be related to real abrupt and sustained variations in the mortality risk. However, it is not always straightforward for a data producer to obtain a broad overview of past coding process methods in the producer's country. The reasons for the occurrence of some of the oldest jumps may have been lost. Therefore, the decision to take into account or not any detected jump that is not confirmed by the data producer will depend on the degree of confidence that the jump is not attributable to a production change.

Some jumps are of great amplitude (e.g., rheumatic heart disease). This may be observed when the cause considered is highly likely to be the result of other causes [10,23]. In that case, the death count time trend is very sensitive to changes in coding rules (e.g., ICD-10 rule 3). However, the absence of high-amplitude jumps is not sufficient to ensure the interpretability of time trends. Time trends for some conditions like hypertension, heart failure, and renal failure have to be interpreted cautiously. Indeed, the approach chosen was to only consider the underlying cause of death, and these specific causes may be selected as underlying, due to lack of additional information about the real underlying cause on the death certificate. In these cases, mortality time trends could be influenced by other conditions or slowly diffused certification changes. A multiple cause approach considering each cause mentioned on the death certificate could bring very different results.

Large jumps may also be observed when a country uses very specific codes. In this study, for practical reasons, it was decided to use the same codes for all the countries. However, the same general method could have been applied to specific codes for each country.

In any event, time trends for causes with large amplitude jumps, even after correction, are to be interpreted with caution.

For some causes, jump amplitude was markedly heterogeneous by age. This result has already been observed in bridge coding studies [11,16]. This result could be attributed to three factors: first, for some causes, subcause structure is different by age, and each subcause is differentially impacted by production change; second, older age mortality is more frequently associated with multiple pathologies, and the selection of one of these as the underlying cause may change with coding rules; third, in certain cases, the same death certificate may be interpreted differently depending on the age of the deceased, and this difference may also depend on the coding rules used.

## Conclusions

The method presented in this paper was successfully applied to a large set of causes of death and countries. The set of causes considered is heterogeneous in terms of frequency of occurrence (e.g., more than a 100-fold difference between the frequencies of cerebrovascular disease and malignant neoplasm of the testes) and sensitivity to coding change (no sensitivity for congenital heart disease and high sensitivity for heart failure).

In the future, it would be of interest to investigate the extent to which such a time series approach could be used in a spatial approach to some specific causes. The hypotheses would then be that a large and clear-cut discontinuous change in cause-specific death count, coinciding with a country's border, is attributable to data production discrepancies rather than to real underlying mortality risk variations.

## Competing interests

The authors declare that they have no competing interests.

## Authors' contributions

All authors read and approved the final manuscript. GR participated in the design of the study, conducted the analysis, and drafted the manuscript. AA and GP conducted the code allocation and revised the manuscript. RH and IP provided the dataset and participated in the revision of the results and manuscript. RW revised the manuscript. EJ and JM participated in the design of the study and revised the manuscript.
